# Bullying and Cyberbullying Perpetration and Victimization: Prospective Within-Person Associations

**DOI:** 10.1007/s10964-022-01704-3

**Published:** 2022-11-17

**Authors:** Antonio Camacho, Kevin Runions, Rosario Ortega-Ruiz, Eva M. Romera

**Affiliations:** 1 Universidad de Córdoba, Córdoba, Spain; 2 The University of Western Australia, Perth, Australia

**Keywords:** Within-person, Longitudinal study, Adolescents, Bullying, Cyberbullying

## Abstract

Bidirectional associations between bullying and cyberbullying have consistently identified during adolescence. However, little is known about how this relationship works on the within-person level, after controlling for potential overlap at the between-person level. This study examined the bidirectional longitudinal associations between bullying and cyberbullying perpetration and victimization during 18-month period over four time points. A total of 2835 participants, aged 11 to 16 years in time 1 (50% girls; *M*_age_ = 13.13, *SD* = 1.06) were surveyed. Random intercept cross-lagged analyses revealed the stability of bullying perpetration and victimization. Cyberbullying victimization predicted inversely bullying and cyberbullying perpetration. The results indicate spirals of positive long-term associations between bullying (perpetration and victimization) and cyberbullying perpetration but no long-terms spirals of victimization.

## Introduction

Bullying has been defined as repeated and intentional aggression by one or more individuals against the victim who is unable to effectively defend himself/herself (Olweus, 1993; Volk et al., 2014). The new realities of information and communication technologies have given rise to cyberbullying, intentional aggressive acts conducted through these technologies (Kowalski et al., 2014). Researchers have recognized the complexity in discriminating between the two forms of violence in adolescents to the point that an outstanding question in the literature is the extent of overlap between bullying and cyberbullying. These questions of overlap are important to understand the developmental sequencing of bullying involvement - as perpetrator and as target of bullying - over time. Questions of overlap also get at issues of role continuity (e.g., are young people who engage in cyberbullying more likely to become perpetrators of offline bullying?) and role inversion (e.g., are targets of bullying more likely to become perpetrators of bullying?). Given the negative impact on psychosocial adjustment of involvement over time in these phenomena there are some research questions that need to be addressed. Is there stability of involvement over time? Does a particular experience provide an ‘entry point’ into further bullying involvement? Does being a target of bullying lead to perpetration? Does traditional bullying perpetration tend to migrate to the online setting, or does cyberbullying prepare students for more direct acts of bullying? Definitive answers to these questions have eluded researchers to date, and (largely unrecognized) limitations of the common methodologies pose severe challenges to those existing preliminary conclusions held by researchers. Designed effective intervention for adolescents involved in bullying requires a clear natural history of the phenomenon of bullying involvement. This study deploys recent developments in cross-lagged panel modeling to provide a close examination of bullying perpetration and victimization both online and off over time, enabling conclusions that do not conflate inter-individual change over time (relative to one another) and intra-individual change (relative to oneself). Given that bullying (about the first years of middle school) and cyberbullying (about the last years of middle school) peak during adolescence (Kowalski et al., 2014; Pabian & Vandebosch, 2016), the present study was a four-wave panel study among middle school adolescents aged 11-16 which tends to peak in around middle adolescence (Kowalski et al., 2014).

### Stability, Role Continuity and Role Inversion in Online and Offline Bullying Involvement

An overlap between bullying and cyberbullying has been mainly established by correlational cross-sectional studies (Baldry et al., 2017; Del Rey et al., 2012; Kowalski et al., 2014; Modecki et al., 2014). A meta-analysis of longitudinal studies provided estimates of the association from victimization to subsequent perpetration and perpetration to subsequent victimization (Walters, 2021). But the estimates from this meta-analysis are based on correlations analyses. Researchers seeking to understand possible causal influences on behavior are often interested in cross-lagged path modeling (CLPM), that estimates concurrent, autoregression and lagged associations. Based on extant research using CLPM, autoregressive paths have shown that online and offline perpetration and victimization are relatively stable over time (Camacho et al., 2021; Giumetti et al., 2022; Pabian & Vandebosch, 2016). These studies have also provided estimates of significant lagged associations between perpetration and victimization conducted online and offline. Research on different aspects of bullying (e.g., bullying and cyberbullying; perpetration and victimization) has developed two lines of hypothesizing: *role continuity* and *role inversion*.

In the *role continuity hypothesis*, involvement in bullying continue and extend it via cyberbullying (Baldry et al., 2016). Based on research to date, adolescents involved in bullying perpetration are more likely to engage in cyberbullying perpetration later (Chu et al., 2018; Giumetti et al., 2022; Pabian & Vandebosch, 2016). Similarly, those involved in cyberbullying perpetration have also been found to have greater subsequent involvement in offline perpetration (Pabian & Vandebosch, 2016). Similarly, higher levels of bullying victimization predict subsequent increased cyberbullying victimization (Giumetti et al., 2022; Pabian & Vandebosch, 2016), and vice versa (Chu et al., 2018; Pabian & Vandebosch, 2016).

*Role inversion* indicates a process in which those involved in one role are more likely to become involved in the other role: victimization may subsequently become perpetrators, and vice versa (Falla et al., 2022; Lee et al., 2021; Pabian & Vandebosch, 2016). Perpetrators of bullying may unknowingly make enemies of people more powerful than themselves, thereby becoming targets of bullying (Malamut et al., 2022). Victims of bullying may come to learn that perpetration is a path toward perceived popularity (if not peer acceptance; Strindberg et al., 2020) and begin bullying other less powerful them themselves. Others may desire revenge but find retaliation ‘in kind’ too difficult due to imbalances in that modality of power (e.g., physical, psychological, and social); consequently, the victims may seek a modality where they are not weak. For example, young people who experience face-to-face victimization may use the Internet to bully others and take revenge on those perceived as bullying them at school (Chu et al., 2018; Runions et al., 2018). The Internet is potentially significant for adolescents who perceive themselves as less empowered, as power imbalance becomes less prominent in the cyberspace which provides other factors such as anonymity or the technical skills to react to damaging interpersonal experiences in the offline context. An example of this is the finding that adolescents with higher levels of bullying perpetration are at increased subsequent risk of cyberbullying victimization (Chu et al., 2018).

### Addressing Methodological Limitations to Differentiate Between- and Within-Person Change

To date, most studies examining these hypotheses have used methodological strategies without a capacity to differentiate between inter-individual change and intra-individual change. Specifically, CLPM has important methodological concerns as it cannot account for trait-like (time-invariant) and state-like (time-variant) individual differences (Hamaker et al., 2015). Data simulation studies have shown that this limitation can result in inaccurate models and thus mistaken conclusions regarding the existence of a lagged path from one variable to another or may result in incorrect estimates of the direction of the causal relationship (Hamaker et al., 2015). Thus, CLPM may indicate a significant positive path from one variable at one time to another variable at the subsequent time, but the real relationship may in fact be negative. Obviously, inaccuracies of this scale will lead to the wrong conclusions regarding causal processes. For an accurate insight into the causal directionality of effects, a differentiation of the between- and within-person level over time is needed. Hamaker et al. (2015) have proposed the random intercept cross-lagged panel model (RI-CLPM), which account for the individual differences by distinguishing among between- and within-person level. By incorporating random intercepts, the RI-CLPM is able to avoid spurious findings regarding the presence of any causal relationships, the temporal priority (and hence likely causal priority) of different variables, and the direction / sign of the estimated lagged relationship (Hamaker et al., 2015).

To date, few studies of bullying and cyberbullying have addressed the influence between perpetration and victimization without confounding inter- and intra-individual. The stability of traditional (offline) bullying victimization and perpetration (autoregressive paths) has been reported consistently positive in both CLPM and RI-CLPM (Davis et al., 2022; Pabian & Vandebosch, 2016; Romera et al., 2021; Zhu et al., 2022). However, the stability apparent in cyberbullying perpetration and cyberbullying victimization has been based on studies using traditional CLPM (Camacho et al., 2021; Giumetti et al., 2022). Two studies using RI-CLPM (Boer et al., 2021; Erreygers et al., 2018) have not replicated this stability, which suggests that the evident continuity of cyberbullying involvement may be a spurious finding based on flawed treatment of variance. This finding - that bullying involvement is relatively stable, whereas cyberbullying involvement is more sporadic- may be based on the specific characteristics of cyberbullying. In cyberbullying, the aggression not necessarily has to be repeated over time by the same person (resending videos or images by others is a way of repeating the aggressive action), there is a higher probability to disengagement chances, and also there is not a clear interdependence between the victim and the aggressor (Huang et al., 2022).

Cross-lagged associations between perpetration and victimization, and the possibility of role inversion, have also been examined using RI-CLPM. Zhou et al. (2022) examined prospective reciprocal associations between perpetration and victimization at the within-person level in a sample of children. Consistent with previous findings in CLPM, they found that an increase from T3 in bullying victimization compared with their average in the study predicted higher involvement in bullying perpetration at time 4. Moreover, bullying perpetration at time 4 also positively predicted bullying victimization in time 5. These findings suggest that once the between- and within-person variance in victimization and perpetration are disaggregated, bidirectional relationships exist. Examining cyberbullying over three-waves six months apart, Erreygers et al. (2018) also explored the bidirectional association between perpetration and victimization for adolescents. In contrast to previous findings using CLPM (Akgül & Artar, 2020; Camacho et al., 2021), they did not find significant association between cyberbullying victimization and subsequent changes in cyberbullying perpetration. The cross-lagged path from cyber-perpetration to cyber-victimization was also not significant. This provides important information on the possible causal development sequencing of cyberbullying involvement. To date, however, no studies have examined the interrelationships of *both* online and offline bullying perpetration and victimization, and thus testing the role continuity and role inversion hypotheses.

## Current Study

Although extensive evidence concerning the association between traditional and cyberbullying perpetration and victimization has been reported, almost all prior studies have used statistical approaches that fail to account for within-person variability (such as CLPM). The dynamic processes of adolescent bullying and cyberbullying involvement at the intra-individual level remain unclear. To address this gap, the aim of the present study was to explore the bidirectional longitudinal associations between offline and online bullying perpetration and victimization using RI-CLPM that accounts for within-person processes. Based on prior research, it was expected that offline bullying perpetration and victimization, and cyberbullying perpetration and victimization would show positive between-individual associations (Hypothesis 1), such that individuals who score higher than their peers on one at one time will tend to score higher at subsequent points. At within-individual stability, it was expected that involvement in offline bullying perpetration and victimization would be stable over time, but cyberbullying would not (Hypothesis 2). Based on the cross-lagged paths and the *role continuity hypothesis*, it was predicted that online and offline bullying perpetration would be reciprocally influenced over time (Hypothesis 3) and bullying and cyberbullying victimization would also show significant bidirectionality over time (Hypothesis 4) (e.g., an increase in bullying perpetration at one time relative to their average across the four time points would be associated with higher levels at a later time in cyberbullying perpetration and vice versa). Finally, according with *role inversion hypothesis*, it was expected that offline victimization and perpetration would be bidirectionally positive associated over time, but not cyberbullying perpetration and victimization (Hypothesis 5). It was also expected that bullying victimization would predict cyberbullying perpetration (Hypothesis 6) and bullying perpetration would be associated with later cyberbullying victimization (Hypothesis 7).

## Methods

### Participants and Procedure

The data were drawn from a larger longitudinal study focusing on characteristics associated with the bullying and cyberbullying, using a sample of adolescents in secondary schools in Southern Spain. The study was approved by the Ethical Committee of the institution of the Spanish authors. To recruit participants, schools were invited to collaborate and were informed of the purpose of the project. Once the management team of each school agreed to participate, parental consent was obtained (5% of parents did not consent for their children to participate in the study). The convenience sample comprised 2835 adolescents (50% girls) between 11- and 16-years old attending Grades 7–9, recruited in 13 middle schools. Data collection occurred during school hours via trained psychologists with research experience. Participants were provided the purpose of the study and standardized information on the study and their participation, emphasizing the voluntary, confidential, and anonymous nature of the data collection. The procedure was identical at each time point. Participants completed the paper-and-pencil questionnaire in approximately 30 min. Adolescents were assessed four times in 18 months (six months after each time point). For the purpose of linking surveys over time, participants were instructed to develop their own personal code with the initial letters of their name and date of birth, so that only they would know this identification and data anonymity would be guaranteed. In the baseline, the mean age of students in November 2017 (Time 1; T1) was 13.10 years (*SD* = 1.06). The socio-demographic characteristics for each time point are shown in Table 1. The average response rate was 88%. Sample attrition was principally due to students absent from school on the day of data collection or having moved schools. Due to the longitudinal design of the study, a test was performed to examine whether non-participation might be associated with any of the study variables. Through logistic regression, no significant differences were reported on the basis of gender, age, cyberbullying and bullying (both perpetration and victimization; at each time) predicting higher or lower participation over time (all *p*s > 0.05).

**Table 1:** Sample distribution

	*N*	Time 1	Time 2	Time 3	Time 4
		November 2017	May 2018	November 2018	May 2019
*n* (p.r.)	2835	2657 (94%)	2515 (89%)	2461 (87%)	2357 (83%)
Gender					
Girls	50%	50%	50%	51%	51%
Boys	50%	50%	50%	49%	49%
Age (SD)		13.10 (1.06)	13.60 (1.12)	14.01 (1.05)	14.54 (1.06)
School					
Rural	52%	52%	52%	52%	53%
Urban	48%	48%	48%	48%	47%
Grade					
1		35%	35%	3%	2%
2		34%	34%	36%	37%
3		31%	31%	32%	31%
4				29%	29%

*p.r.* participation rate

### Measures

#### Bullying

The *European Bullying Intervention Project Questionnaire* (Ortega-Ruiz et al., 2016) was applied to measure bullying victimization and perpetration. The questionnaire included 14 items, 7 for each subscale: victimization (e.g., “Someone has said mean things about me to other people”) and perpetration (e.g., “I have stolen or broken someone’s things”). Before completing the questionnaire, participants were provided the characteristics of bullying (intentionality, power imbalance, repetition over time) to differentiate it from other aggressive behaviors. The frequency of the adolescents’ behaviors was addressed with a five response options as *never* (0), *once or twice* (1), *once or twice a month* (2), *about once a week* (3), and *more than once a week* (4), and continuous scores were used for analyses. For each time, internal reliability using McDonald’s omega was 0.86, 0.86, 0.86 and 0.85 for victimization and 0.81, 0.82, 0.81 and 0.77 for perpetration, respectively. The confirmatory factor analysis showed good psychometric properties of the two-factor structure, as proposed in the original study, with the current sample at T1: *χ*^2^ = 678.430, *df* = 76, *p* < 0.001; CFI = 0.958, TLI = 0.949, RMSEA = 0.055, 90% CI [0.051, 0.059], SRMR = 0.064.

#### Cyberbullying

The *European Cyberbullying Intervention Project Questionnaire* (Ortega-Ruiz et al., 2016) was applied to measure cyberbullying victimization and perpetration. The questionnaire included 22 items, 11 for each subscale: victimization (e.g., “Someone threatened me through texts or online messages”) and perpetration (e.g., “I posted personal information about someone online”). Adolescents were asked to the frequency of cyberbullying from 0 (*never*) to 4 (*more than once a week*). For each time, McDonald’s omega was 0.87, 0.85, 0.88 and 0.89 for victimization and 0.87, 0.89, 0.89 and 0.89 for perpetration, respectively. The confirmatory factor analysis showed good psychometric properties of the two-factor structure, as proposed in the original study, with the current sample at T1: *χ*^2^ = 1085.311, *df* = 288, *p* < 0.001; CFI = 0.956, TLI = 0.952, RMSEA = 0.040, 90% CI [0.038, 0.043], SRMR = 0.067.

#### Covariates

Age and gender were collected as socio-demographic information to address any differences in variables based on these characteristics.

### Data Analytic Strategy

Spearman correlations and the Intraclass Correlation Coefficient were performed in preliminary analyses. To analyze the prospective relationships between bullying and cyberbullying by disaggregating the between- and within-person variance a random intercept cross-lagged panel model (RI-CLPM; Hamaker et al., 2015) was estimated using *Mplus* 8.7 (Muthén & Muthén, 1998). The between-person level (time-invariant characteristics) captures the stability in each construct through the random intercept that is estimated with the score across the four times. The between-person level includes covariances between the random intercepts of variables of the study. The variance at the within-person level (time-variant characteristics) is captured by the participants’ time-to-time deviation from the individual expected score. These analyses permit conclusions regarding whether previous increases or decreases of face-to-face and online victimization and perpetration from their own average level across the four time points are associated with subsequent changes in bullying and cyberbullying involvement. The within-person level includes autoregressive paths (e.g., cyberbullying victimization at T1 on cyberbullying victimization at T2), cross-lagged paths (e.g., bullying victimization at T1 on cyberbullying perpetration at T2), covariances between variables in T1 (e.g., cyberbullying perpetration at T1 with bullying perpetration at Time 1), and the residual covariances of the variables at T2, T3 and T4. Gender and age were included in the model as time-invariant predictor of observed variables, as boys have been found to have a higher prevalence of involvement in perpetration (Smith et al., 2019), while middle adolescents tend to have higher involvement in cyberbullying (Camacho et al., 2021).

The data were tested for missingness. Little’s MCAR (Little, 1988) test was significant (*p* < 0.001) indicating that the data were not missing completely at random (MCAR). Based on the low normed chi-square (*χ*^2^/*df* = 1.79) the data were deemed to be missing at random (MAR) (Bollen, 1989). Therefore, missing data were addressed with full information maximum likelihood (FIML). Maximum likelihood estimation with robust standard errors (MLR) was used to address the non-normally distributed nature of the variables. For optimal standard model fit indices, comparative fit index (CFI) should be above 0.90, and root mean square error of approximation (RMSEA) should not exceed 0.08 (Hu & Bentler, 1999). Because the adolescents were placed in schools, the command “type = complex” was used to handle the clustering effects on standard errors.

The associations between the study variables were analyzed through a set of models with the aim to choose the most parsimonious model whose change in model fit was not significant (Kline, 2015). First, an unconstrained model (model 1) where the components of the RI-CLPM were freely estimated. Then, a stepwise series of constraints were added to match the paths over time: autoregressive paths (model 2), cross-lagged paths (model 3) and correlated changes within-time (model 4). Significant differences between model fit comparisons were considered when two of the following criteria were attained: chi-square difference test at *p* < 0.05 (Satorra & Bentler, 2001), ∆CFI ≥ 0.01 and ∆RMSEA ≥ 0.015 (Chen, 2007). In the lack of differences between the nested models, the model with the most constraints was retained.

## Results

### Preliminary Analyses

Descriptive statistics and Spearman’s bivariate correlations among study variables are reported in Table 2. The cross-sectional and longitudinal association between bullying and cyberbullying perpetration and victimization were low-moderate positive.

**Table 2:** Spearman’s bivariate correlations and descriptive statistics of study variables

	1	2	3	4	5	6	7	8	9	10	11	12	13	14	15	16
1. Bullying perpetration T1	–															
2. Bullying perpetration T2	0.51	–														
3. Bullying perpetration T3	0.42	0.47	–													
4. Bullying perpetration T4	0.40	0.45	0.50	–												
5. Bullying victimization T1	0.55	0.33	0.28	0.29	–											
6. Bullying victimization T2	0.31	0.53	0.29	0.29	0.52	–										
7. Bullying victimization T3	0.28	0.32	0.55	0.35	0.42	0.49	–									
8. Bullying victimization T4	0.25	0.29	0.33	0.56	0.40	0.45	0.54	–								
9. Cyberbullying perpetration T1	0.47	0.37	0.31	0.33	0.34	0.22	0.21	0.21	–							
10. Cyberbullying perpetration T2	0.38	0.52	0.35	0.34	0.22	0.30	0.21	0.18	0.44	–						
11. Cyberbullying perpetration T3	0.31	0.39	0.50	0.38	0.18	0.21	0.31	0.22	0.43	0.47	–					
12. Cyberbullying perpetration T4	0.32	0.33	0.39	0.50	0.18	0.16	0.25	0.29	0.36	0.40	0.48	–				
13. Cyberbullying victimization T1	0.42	0.30	0.27	0.29	0.53	0.36	0.34	0.32	0.59	0.36	0.32	0.29	–			
14. Cyberbullying victimization T2	0.29	0.41	0.28	0.28	0.33	0.46	0.33	0.33	0.34	0.57	0.39	0.34	0.48	–		
15. Cyberbullying victimization T3	0.26	0.33	0.38	0.33	0.31	0.36	0.49	0.37	0.34	0.37	0.60	0.37	0.44	0.54	–	
16. Cyberbullying victimization T4	0.25	0.28	0.31	0.43	0.31	0.32	0.37	0.50	0.32	0.31	0.37	0.60	0.42	0.45	0.50	–
*M*	0.25	0.28	0.20	0.21	0.55	0.56	0.40	0.42	0.14	0.14	0.12	0.12	0.23	0.21	0.20	0.20
SD	0.42	0.45	0.38	0.36	0.70	0.67	0.58	0.56	0.34	0.36	0.32	0.31	0.43	0.37	0.39	0.38
Range	0–4	0–4	0–4	0–4	0–4	0–4	0–4	0–4	0–4	0–4	0–4	0–4	0–4	0–4	0–4	0–4

All correlations were significant at *p* < 0.001

In relation to the Intraclass Correlation Coefficient, the within-person level variance in each measure was higher than 10% (58% for bullying perpetration; 49% for bullying victimization; 63% for cyberbullying perpetration; 54% for cyberbullying victimization). This warrants use of RI-CLPM to disaggregate the between- and within-person variance (Hamaker et al., 2015).

### Random-Intercept Cross-Lagged Panel Model

Steps were followed to choose the most parsimonious model before analyzing the prospective relationships between the study variables (see Table 3). In model 1, paths were allowed to vary over time. This unconstrained model showed an excellent model fit. In model 2, the autoregressive paths were constrained to be equivalent over time. This model was not significantly different compared to the unconstrained model since at least two of the model fit comparative criteria were not violated (see Table 3). In model 3, imposing further constraints on cross-lagged paths did not result in significant differences compared to model 2. For model 4 within-time correlated changes were constraining, with no significant differences in model fit compared to model 3. Thus model 4 was retained as the most parsimonious to explore the prospective association between bullying and cyberbullying.

**Table 3:** Model fit statistics of random intercept cross-lagged panel model

Model	Model fit				Model fit comparison		
	*χ*^2^	df	CFI	RMSEA [90% CI]	∆*χ*^2^ (∆df)	∆CFI	∆RMSEA
Model 1	48.320	38	0.998	0.010 [0.000, 0.018]	–	–	–
Model 2	80.170**	46	0.994	0.017 [0.010, 0.023]	24.192 (8)**	0.004	0.007
Model 3	120.834***	70	0.991	0.017 [0.011, 0.021]	40.605 (24)*	0.003	0.000
Model 4	126.449**	82	0.992	0.014 [0.009, 0.019]	14.496 (12)	0.001	0.003

*CFI* comparative fit index, *CI* confidence interval, *RMSEA* root mean square error of approximation

*p < 0.05; **p < 0.01; ***p < 0.001

**Fig. 1:**
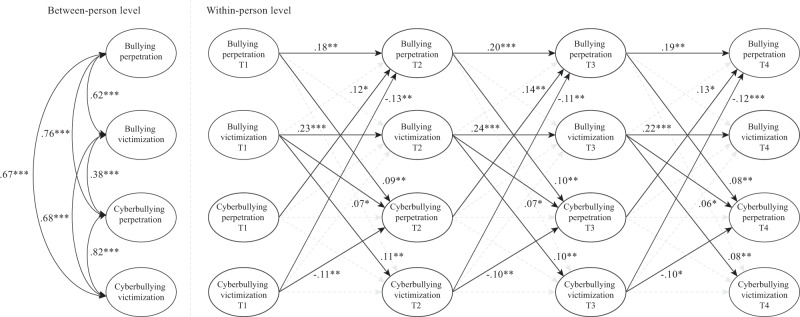
Random intercept cross-lagged panel model. The within-time covariances and the non-significant autoregressive and cross-lagged paths at the within-person level are not illustrated for simplicity. These associations are reported in Table 4. *p < 0.05; **p < 0.01; ***p < 0.001

The results of the RI-CLPM were reported in STDYX standardized estimates (see Fig. 1). At the between-person level, the significant positive covariances between random intercepts of bullying and cyberbullying perpetration and victimization suggest that adolescents who reported higher levels of one variable at the four time points also reported higher levels in other variables compared to other adolescents (Hypothesis 1). At the within-person level, significant and positive autoregressive paths for both bullying perpetration and victimization indicate that adolescents who report a higher-than-expected score are likely to report a higher-than-expected score on subsequent times (Hypothesis 2). Notably, the autoregressive paths of cyberbullying perpetration and victimization both were not significant (see Table 4; Hypothesis 2). In relation to cross-lagged paths, positive cross-lagged reciprocal effects were found across time between bullying and cyberbullying perpetration (Hypothesis 3). Offline bullying victimization predicted subsequent cyberbullying victimization (Hypothesis 4), providing support for the *role continuity hypothesis*. With regard to the *role inversion hypotheses* (Hypotheses 5-7), significant negative paths from online victimization to both bullying and cyberbullying perpetration were discovered (Hypotheses 5 and 7), while face-to-face victimization predicted subsequent increases in cyberbullying perpetration (Hypothesis 6). Regarding the association between variables within-time, the four study variables were positively associated (see Table 4).

**Table 4:** Standardized coefficients of within-person level results in RI-CLPM

Covariances	Time 1	Time 2	Time 3	Time 4
Bullying perpetration ↔ Bullying victimization	0.41***	0.41***	0.52***	0.55***
Bullying perpetration ↔ Cyberbullying perpetration	0.46***	0.42***	0.52***	0.52***
Bullying perpetration ↔ Cyberbullying victimization	0.36***	0.33***	0.36***	0.35***
Bullying victimization ↔ Cyberbullying perpetration	0.31***	0.25***	0.30***	0.30***
Bullying victimization ↔ Cyberbullying victimization	0.52***	0.35***	0.39***	0.37***
Cyberbullying perpetration ↔ Cyberbullying victimization	0.63***	0.67***	0.71***	0.65***
Autoregressive path	T1 → T2 *ß* (SE)	T2 → T3 *ß* (SE)		T3 → T4 *ß* (SE)
Bullying perpetration → Bullying perpetration	0.18** (0.06)	0.20*** (0.05)		0.19** (0.06)
Bullying victimization → Bullying victimization	0.23*** (0.04)	0.24*** (0.04)		0.22 (0.04)
Cyberbullying perpetration → Cyberbullying perpetration	0.08 (0.06)	0.08 (0.07)		0.08 (0.07)
Cyberbullying victimization → Cyberbullying victimization	−0.01 (0.08)	−0.01 (0.06)		−0.01 (0.06)
Cross-lagged path	T1 → T2 *ß* (SE)	T2 → T3 *ß* (SE)		T3 → T4 *ß* (SE)
Bullying perpetration → Bullying victimization	0.00 (04)	0.00 (04)		0.00 (04)
Bullying perpetration → Cyberbullying perpetration	0.09** (0.03)	0.10** (0.03)		0.08** (0.03)
Bullying perpetration → Cyberbullying victimization	0.02 (0.04)	0.02 (0.04)		0.02 (0.03)
Bullying victimization → Bullying perpetration	0.00 (04)	0.00 (04)		0.00 (04)
Bullying victimization → Cyberbullying perpetration	0.07* (0.04)	0.07* (0.04)		0.06* (0.03)
Bullying victimization → Cyberbullying victimization	0.11** (0.04)	0.10** (0.03)		0.08** (0.03)
Cyberbullying perpetration → Bullying perpetration	0.12* (0.05)	0.14** (0.05)		0.13* (0.05)
Cyberbullying perpetration → Bullying victimization	0.04 (0.04)	0.05 (0.05)		0.05 (0.04)
Cyberbullying perpetration → Cyberbullying victimization	−0.07 (0.07)	−0.07 (0.07)		−0.06 (0.06)
Cyberbullying victimization → Bullying perpetration	−0.13** (0.04)	−0.11** (0.04)		−0.12** (0.04)
Cyberbullying victimization → Bullying victimization	−0.02 (0.05)	−0.02 (0.04)		−0.02 (0.05)
Cyberbullying victimization → Cyberbullying perpetration	−0.11** (0.05)	−0.10** (0.04)		−0.10* (0.04)

*p < 0.05; **p < 0.01; ***p < 0.001

Based on time-invariant predictors, boys show higher involvement than girls in both bullying and cyberbullying perpetration, as in online and face-to-face victimization at T1 (see Table 5). Older participants in the study showed higher involvement than younger participants on bullying perpetration (at T1 and T2), cyberbullying perpetration (from T1 to T3) and cyberbullying victimization (from T1 to T4) (see Table 5).

**Table 5:** Standardized coefficients of the time-invariant predictors in RI-CLPM

	Gender^a^		Age	
	*ß*	SE	*ß*	SE
Bullying perpetration T1	−0.13***	0.02	0.04*	0.03
Bullying perpetration T2	−0.12***	0.03	0.04**	0.01
Bullying perpetration T3	−0.12***	0.03	0.01	0.01
Bullying perpetration T4	−0.10***	0.02	0.01	0.01
Bullying victimization T1	−0.11**	0.03	0.01	0.02
Bullying victimization T2	−0.03	0.04	−0.04	0.02
Bullying victimization T3	−0.03	0.02	−0.03	0.03
Bullying victimization T4	−0.03	0.02	−0.03	0.02
Cyberbullying perpetration T1	−0.08***	0.02	0.05***	0.01
Cyberbullying perpetration T2	−0.06**	0.02	0.11***	0.02
Cyberbullying perpetration T3	−0.05*	0.02	0.10***	0.02
Cyberbullying perpetration T4	−0.05**	0.02	0.04	0.03
Cyberbullying victimization T1	−0.05*	0.02	0.05***	0.01
Cyberbullying victimization T2	−0.01	0.02	0.04***	0.01
Cyberbullying victimization T3	−0.02	0.02	0.04***	0.01
Cyberbullying victimization T4	−0.02	0.02	0.02*	0.01

*p < 0.05; **p < 0.01; ***p < 0.001

^a^Gender was coded as: 0 = boy and 1 = girl

## Discussion

Research has suggested the *role continuity and inversion hypotheses* about the phenomena of bullying and cyberbullying on the involvement of adolescents in perpetration and victimization. However, the bidirectional influence association has mainly focused on approaches that may be misleading because associations within- and between-person were not disaggregated. This study used cutting-edge within-person statistics to correct for limitations with traditional modeling that may have led to spurious findings. The present research captured intra-individual longitudinal fluctuations between bullying perpetration, bullying victimization, cyberbullying perpetration, and cyberbullying victimization over time after controlling for time-invariant associations at the between-person level by using RI-CLPM.

The overlap between bullying perpetration, bullying victimization, cyberbullying perpetration and cyberbullying victimization were reported by the positive associations at a stable *between-person* level (Hypothesis 1). These moderate/strong correlations of random intercepts indicated an overall time invariance of adolescent bullying involvement of adolescents. Consistent with previous cross-sectional research (Baldry et al., 2017; Del Rey et al., 2012), higher involvement in one aspect of bullying across the four time points (e.g., offline victimization) was associated with high involvement in the other aspects (e.g., cyberbullying perpetration) overall. Associations strong overall (ranging from *r* of 0.68–0.82) except for the association of bullying victimization and cyberbullying perpetration, which had a notably more modest association. However, these findings do not clarify which features are antecedent or consequence.

### Stability of Bullying and Cyberbullying

Once the stable differences between individuals have been controlled for, a more accurate understanding may be provided on influence of time-variant variables and likely causal processes involved. In support of Hypothesis 2 and consistent with previous research (Boer et al., 2021; Cogo-Moreira et al., 2021; Davis et al., 2022; Romera et al., 2022), involvement in perpetration and victimization was stable for offline bullying, but not in cyberbullying. Such findings for offline bullying are consistent with those studies even when CLPM was employed (and thus without separating between- and within-person variance; Chu et al., 2018; Pabian & Vandebosch, 2016). Using RI-CLPM, the findings of the present study indicate that there is no direct stability in cyberbullying involvement over time (Boer et al., 2021; Erreygers et al., 2018), in contrast with previous research using CLPM (Camacho et al., 2021; Giumetti et al., 2022). The stability over time of victimization and perpetration may be supported by the hierarchical nature of offline bullying as a group process. However, the involvement of adolescents in cyberbullying tends to have a more sporadic and less sustainable character over time (Huang et al., 2022; Smahel et al., 2020). The role of group dynamics in the relative stability of offline bullying remains to tested more directly but may speak to a more complex developmental phenomenon than current models of role inversion and continuity (see below).

### Continuity of Perpetration and Victimization across Bullying and Cyberbullying

Although cyberbullying perpetration was not stable per se in these analyses, there was a stable pattern of bidirectionality between online and offline bullying at the within-person level, wherein at each wave, cyberbullying was predicted by subsequent offline bullying and went on to predict the later increases in offline bullying perpetration, as per Hypothesis 3. This provides a more conservative replication of previous studies that used CLPM (Chu et al., 2018; Giumetti et al., 2022; Pabian & Vandebosch, 2016). According to the *role continuity hypothesis* (Baldry et al., 2016), adolescents are expected to adopt the same patterns across phenomena. The *co-construction theory* has been applied to account for how adolescents construct their digital social interactions in a similar way to their non-digital environment (Subrahmanyam et al., 2006). The online phenomenon may spread face-to-face perpetration by attacking others on the Internet to further increase the damage. Regarding the opposite influence, after online perpetration, adolescents may tend to endorse surrounding states or normative beliefs that their later involvement in bullying perpetration is an acceptable behavior (Wright & Li, 2013).

Bullying and cyberbullying victimization were also expected to be bidirectionally associated (Hypothesis 4). In line with previous research (Giumetti et al., 2022; Pabian & Vandebosch, 2016), offline bullying victimization predicted cyberbullying victimization, suggesting that victimization may begin in person but migrate to online settings. Among the possible underlying assumptions for this continuity in victimization are the spread of offline interpersonal relationships through the Internet and the *victim schema model*. As the Internet during these ages is a setting where peers extend their face-to-face interpersonal relationships, the perpetrator may spread online to enhance the potential for harming the victim (Wright & Li, 2013). In addition, *victim schema models* (Rosen et al., 2007) may also contribute to role continuity, as experience of being a target in one setting may generate negative cognitive biases and maladaptive coping in peer relationships (Camacho et al., 2022), that lead to greater perceptions of threat, mistrust and increased likelihood of victimization both offline and virtual phenomena (Chu et al., 2018; Rodríguez-de Arriba et al., 2022). However, unlike previous studies in CLPM (Chu et al., 2018; Pabian & Vandebosch, 2016), when differences in stable victimization at between-person level were controlled for, an increase in online victimization within-person level did not predict a higher involvement in face-to-face victimization. Previous studies on internalizing problems arising from victimization have also found differences between online and face-to-face victimization. While studies have reported that both bullying and cyberbullying victimization are subsequently associated with greater depressive symptoms (Fredrick et al., 2022; He et al., 2022), such associations are consistent when analyzed at the within-person level in bullying (Li et al., 2021), but not in cyberbullying (Boer et al., 2021). Such divergences could be explained by the role of the online context for adolescents. In cyberbullying, any person may be exposed to be a victim even those with greater resilience and adaptative coping strategies that, in contrast, can be effective to avoid face-to-face victimization.

### Inversion of Victimization and Perpetration across Bullying and Cyberbullying

According to the *role inversion hypothesis* (Mishna et al., 2012), those who become involved in one role (target or perpetrator of bullying) are more likely to take on the other role later. Counter to the fifth hypothesis and in contrast to previous research (Zhou et al., 2022), changes in offline victimization yielded no direct deviations in offline bullying perpetration at any wave. However, there was evidence of a more indirect path whereby being targeted offline was positively associated with subsequent cyberbullying perpetration (providing support for Hypothesis 6 and consonant with Chu et al., (2018)), which in turn was positive associated with face-to-face perpetration. This suggests that targeted adolescents may use the more covert cyber setting as an incubator for bullying perpetration and then move on – perhaps with growing confidence – to engage in overt face-to-face bullying. As Ybarra and Mitchell (2004) have conjectured, for victims of offline bullying, the Internet may provide a setting for dominating others as compensation for their own social position. Such a contrast could be based on the helplessness of the victim in the face-to-face situation. The power imbalance provides the perpetrator a safe position, as the victim may not have enough physical and psychological strengths to overcome the social gap and address the perpetrator with revenge. However, the power imbalance differs in the online phenomenon due to anonymity and disinhibition. The difficulty to identify the perpetrator and the avoidance of retaliation may develop into the perception of a lack of responsibility and deindividualization of the behavior. This may provide the victim of bullying with the courage to engage in cyberbullying perpetration later as a means of revenge (Runions et al., 2018) or as a bid to obtain power or social status, which they may have attributed to their own bullies in their past experiences.

In the present study, it was also expected that bullying perpetration would be associated with subsequent cyberbullying victimization (Hypothesis 7). In contrast with two previous studies (Lee et al., 2021; Pabian & Vandebosch, 2016), face-to-face perpetration did not predict online victimization, while an increase in cyberbullying victimization predicted decreases in cyber- and offline bullying perpetration, suggesting that experiences of cyber-victimization may protect against future bullying perpetration both offline and online at the individual level. This finding, in contrast to the significant positive pathways from offline victimization to cyberbullying perpetration, presents a paradox. Once between-person variance is accounted for, why would an increase in face-to-face victimization lead to an increase in the involvement in online perpetration, whereas online victimization leads to a subsequent decrease in perpetration, online and off?

Compared to online victimization, offline victimization is less strongly associated with increased internalizing problems (e.g., fear, humiliation, anxiety or depression; Dennehy et al., 2020). Being cyberbullied, with the constant access to the victim (24 h a day, 7 days a week) and the permanency of the evidence of bullying existing on the Internet (see Runions et al., 2013 for a review) could generate in the victim a feeling of chronic vulnerability, helplessness, and powerlessness to address the revenge through perpetration. These processes may lead individuals to become aware of the harm caused by perpetration, as well as experiencing the emotions of others, resulting in a decreased likelihood of subsequent online aggression towards others (see meta-analysis, Kowalski et al., 2014). An adolescent who experiences victimization for a limited period may develop some sensitivity to the aggressive behaviors of bullying, knowing personally the associated psychological consequences.

An alternate possibility is that targets of cyberbullying do not see or project the same accrual of social status (e.g., social impact or perceived popularity; Guy et al., 2019) to the perpetrator as do targets of face-to-face bullying, where the group dynamics are more evident, and where popularity may be attributed to one’s tormentor. The perception that bullying leads to popularity may drive those who are predominantly bullied offline in a way that diverges from the experience of cyberbullied adolescents.

Future research might address this issue through models that examine whether internalizing symptoms or coping strategies with victimization may play a mediating role in the effects of cyberbullying victimization and bullying victimization on perpetration at within-person level.

### Limitations and Practical Implications

Though the study presents certain strengths (large sample, longitudinal data, and within- and between-person level), the findings bear limitations to be addressed in future research. First, given that perpetration and victimization were both treated as discrete variables, the co-occurrence (bully-victim status) could not be modeled. This is unfortunate, as dual involvement in both roles is an important phenomenon that this work is concerned with. Second, only self-report measures were used, which may have increased shared method variance and social desirability bias. This bias could be addressed in future research by using peer or teacher nomination of victims and perpetrators of traditional bullying. Although the repeated-measures time intervals (at the beginning and end of the school year across two school years) provide insight into potential effects over time, given the dynamic social relationships during adolescence, a shorter time frame might capture changes to a better extent, especially in cyberbullying. The sample (between 11 and 16 years at T1; 13-18 at T4) does not allow extrapolation to the whole period of adolescence, nor does it capture specific key developmental periods that may influence the association between variables (e.g., transition from primary to secondary school, pubertal maturational stage, development of romantic relationships). Furthermore, future studies could also consider controlling for factors that could influence the involvement of bullying and cyberbullying (e.g., ethnicity, socioeconomic status, internet use, parental styles, internalizing symptoms, coping strategies). The sample derives from a specific context in the south of Spain with a majority Caucasian population. Future studies could consider more demographically diverse regions with cross-cultural designs to further enhance the relevance of the findings.

Beyond the limitations, the study provides support for psychoeducational strategies to address bullying and cyberbullying. Anti-bullying programs aimed at reducing perpetration and victimization rates in face-to-face phenomenon should target the involvement of schoolchildren in cyberbullying (Casas et al., 2018). It is also important to raise awareness that adolescents who are targets of bullying may come to the erroneous belief that perpetration of bullying is in their best interests. Special emphasis should be focused on adolescents who experience offline bullying since such experience could be extended online, and problem-focused coping strategies to deal with the situation and with attendant emotional problems (e.g., anxiety and depression) may be of value. Such interventions should also address issues of moral sensitivity to adhere to moral standards and decrease the selective deactivation of the moral self-regulation process as a means to avoiding the normalization of violence, which may fuel vicious circles of perpetration and victimization (Romera et al., 2021).

## Conclusions

The association between bullying and cyberbullying (victimization and perpetration) have been accounted from the *role continuity* and *inversion hypotheses*. From a longitudinal approach, the *role continuity hypothesis* addresses how previous involvement in bullying may subsequently extend to cyberbullying phenomena. Whereas the *role inversion hypothesis* addresses how the involvement of adolescents in one phenomenon may lead to the involvement of other behaviors later (first victimization and then perpetration or vice versa). The present study provides a notable contribution to the literature by addressing these hypotheses longitudinally using new statistical approaches that disentangle between- and within-individual variance over time. At the between-person level, stable differences across individuals have highlighted the well-known overlap between bullying and cyberbullying victimization and perpetration. At the within-person level, the longitudinal influence between variables has been established by considering the individual and its temporal evolution. Through the state-like characteristics, the present study considers the influence between the variables considering that the involvement of many adolescents in the phenomena may be spontaneous and connected to a particular situation, which is in line with previous descriptive studies on bullying and cyberbullying. In sum, three conclusions are noteworthy. First, being involved in bullying, as victim or bully, is positively long-term associated to cyberbullying perpetration. Second, there is no long-terms spirals of victimization off and online. Third, having experiences of cybervictimization may predict not being a bully and cyberbully. Taken together, the findings highlight the importance of considering both aggressive behaviors (off y online) to understand and prevent students’ involvement. Effective intervention depends upon a thorough causal understanding of how involvement in bullying arises and evolved over time; this study provides an important step in explicating these processes. Knowledge of interactive play between the diverse manifestations is an important contribution to the shaping of preventive and intervention strategies adapted to the social reality of adolescents in an online and offline world that is interconnected in many of its mechanisms.
